# Evaluation of an Alternative Learning System for youths at risk of involvement in urban violence in the Philippines

**DOI:** 10.1186/s12962-021-00320-5

**Published:** 2021-10-09

**Authors:** Nishant Mehra, Shr-Jie Sharlenna Wang, Juancho Reyes, Mette Møhl Ambjørnsen, Johan Jarl

**Affiliations:** 1 Danish Institute Against Torture, København, Denmark; 2 Department of Clinical Sciences (Malmö), Health Economics Unit, Lund University, Lund, Sweden; 3 Balay Rehabilitation Centre, Quezon, Philippines

**Keywords:** Economic evaluation, Alternative learning system, Psychosocial intervention, Urban violence, Youth, Young people

## Abstract

**Background:**

Globally, violence disproportionately affects young people, leading to injury, hospitalisation, death, social dysfunction, and poor mental wellbeing. Moreover, it has far-reaching economic consequences for whole nations, due to loss of productivity. Research suggests that attaining a higher level of education promotes factors that insulate youths from poverty and violence.

**Purpose:**

In this study, we investigated the outcomes, the cost, and the cost-effectiveness of a non-formal education program with an additional psychosocial component. The short-term outcome measure was an increase in educational attainment, a crucial step for youth empowerment. The program analysed was the Alternative Learning System (ALS) offered by the Balay Rehabiliation Centre in Bagong Silang, an urban slum in Manila, which targeted out of school youth.

**Methods:**

The cost-effectiveness analysis of ALS compared to a ‘do nothing approach’ was performed from the perspective of the service provider. The study sample comprised 239 learners who were enrolled in the ALS during 2015–2018. For the ‘do nothing’ comparator, a counterfactual scenario was hypothesised. The average cost of the intervention per enrolled learner, and the incremental cost effectiveness ratio (ICER) for passing the Accreditation and Evaluation (A&E) exam at elementary or secondary level, were calculated.

**Results:**

The ALS intervention studied resulted in 41% (n = 97) of the learners passing the examination over a period of four years (from 2015–2018). The estimated total cost of the intervention was $371,110, corresponding to $1550 per enrolled learner. The incremental cost-effectiveness ratio for a pass in the exam was found to be $3830. Compared to other, international, alternative learning interventions, the ALS intervention as used in Bagong Silang was found to be more cost-effective.

**Conclusion:**

From the service provider perspective, the ALS for out-of-school young people was found to be a valuable investment to benefit poor young people living in slums in Manila**.**

## Introduction

Globally, violence puts an immense strain on health care expenditure, and has far reaching consequences for national economies, due to loss of productivity and an increase in the expense of law enforcement [1]. Household poverty and neighborhood deprivation are both strongly related to youth violence [2, 3]. Among young people, violence is often used as a tool to gain self-esteem when they are exposed to adversities. This is often seen among people who are forced to live under impoverished circumstances [2]. Violence is a characteristic of the area where our study was performed, Bagong Silang, a Barangay (a large municipal entity) in the city of Manila with a population of approximately 240,000 [4]. It was established in the 1970s as a relocation site for slum-dwellers who had previously lived in different parts of Metro Manila. Since its establishment, there have been immense socioeconomic and political problems in Bagong Silang, associated with experiences of all types of violence and torture [4, 5]. Common problems are lack of employment opportunities, socioeconomic inequalities, teenage pregnancies, child abuse and drug addiction [4]. Balay Rehabilitation Centre (BRC), a non-governmental organization based in Manila supports vulnerable young people who are at risk of involvement in violence. BRC practices psychosocial rehabilitation based on evidence-based community led interventions, such as encouraging nurturing relationships between parents and children, raising awareness on human rights, offering psychosocial counselling services to victims of violence and promoting skill development through alternative educational interventions. Low levels of education has been shown to be a risk factor for poverty and violent behavior, especially among young people [7]. In 2015, around 14% of young people in the age group of 15–29 years living in Manila dropped out of school [8], mainly due to lack of personal interest and the high cost of education [9]. Based on the overview of the empowerment theory applied to youth violence prevention programs, we hypothesized that youths can be empowered to move out of poverty and reduce the risk of exposure to violence by raising their educational level and equipping them with the knowledge and skills necessary for the labor market [10].

An Alternative Learning System (ALS) program was initiated in 2004 by the Department of Education, Government of the Philippines, throughout the country with the aim of providing school drop outs with access to free basic education [11]. In 2015, the national average of the target population enrolled in ALS was less than 10% and in Manila enrollment was around 6% [8].

The ALS program is a non-formal education program that is free of charge for the participants (referred to as "learners" from this point). Enrollment in ALS allows the learners to take the ALS Accreditation and Evaluation (A&E) Exam, offered by the government, where a pass is considered comparable to graduation from the formal education system [12]. Through implementation of different programs utilizing various delivery methods, ALS strives to reach and help learners in ways that fit their distinct needs [11].

### Description of the intervention

The study described here investigated the ALS program as one part of a larger youth empowerment program as planned and run by BRC targeting children, adolescents and young adults exposed to violence in Bagong Silang, Manila. The total population in Bagong Silang is around 245,000 and nearly 19% are aged 16–24 years old [5]. Though the school dropout rate at the national level is about 14%, the rate is likely to be higher in Bagong Silang, around 20%. Therefore, we assume that this ALS targeted around 5000–9800 at-risk youth in Bagong Silang. Our study population accounts for 4–8% of the community population.

The ALS program was advertised in Bagong Silang at the beginning of each academic year and new learners were recruited on a voluntary basis. The learners were first screened to ascertain their educational status, and classified into three levels: Basic Literacy; ALS Elementary; ALS Secondary, this terminology is defined in the International Standard Classification of Education (ISCED) for the formal mode of education [13]. At the time of recruitment, all learners were also interviewed about their experiences of violence in the community. Their psychosocial needs were assessed by individual interviews, focussed group discussions and home visitation to check living conditions and family dynamics. Based on their needs, targeted psychosocial interventions were offered to 22% of learners. These included case management through individual or group counselling sessions, psychoeducational sessions on topics such as anger management, self-esteem and reproductive health, and welfare assistance, including financial support for education and mental healthcare.

Educational sessions providing instruction in six interrelated learning streams (communication skill, scientific literacy and critical thinking, mathematical and problem solving skills, life and career skills, understanding self and society and digital citizenship) are offered as required for the A&E examination [14]. These sessions were offered three times a week at the ALS Centre in Bagong Silang, a small compound rented for conducting ALS teaching sessions and psychosocial activities. In the Basic Literacy program, the sessions lasted two hours, while for ALS Elementary and ALS Secondary lasted 4–5 h. The sessions ran for 10 months per year, from January to October, and at the end learners took the ALS Accreditation and Evaluation (A&E) Exam conducted by the Department of Education [11]. Two ALS instruction managers (teachers) conducted the education sessions. One psychosocial worker managed the psychosocial activities. A project supervisor, a project coordinator, one researcher and an administrative staff supported the smooth implementation and running of the intervention.

The aim of this study was to evaluate the ALS intervention in Bagong Silang in terms of costs, short-term effects (education and employment), and cost-effectiveness from the service provider’s perspective.

## Methods

This study is an evaluation of a real-world intervention and not a trial and baseline characteristics can therefore not be influenced. The study sample included all enrolled in ALS at Bagong Silang during the years 2015–2018. The costs and short-term effects were evaluated during the full period of the ALS program, January 2015 to October 2018. In addition to the implementation and running of the intervention, the period included the recruitment processes and examinations [8].

Data on outcomes was extracted directly from reports obtained from BRC. The primary short-term outcome measure used was educational attainment in terms of achieving an equivalency to formal school education, measured as passing the ALS Accreditation and Evaluation (A&E) examination within the period of the intervention, as this is a well-defined and reproducible outcome [15, 16]. The examination is conducted at two educational levels: Elementary and Secondary, and attaining a pass at either level was considered a successful outcome of the intervention in this study. If a learner managed to pass both elementary and secondary level exams during the period, this was considered as two successful outcomes.

Data on long-term outcomes such as getting a job or a reduction in experience of violence were also collected by BRC through surveys. However, since the intervention only ended in 2018, the data on long-term outcomes was limited for the purpose of the current study. We therefore did not include these outcomes in the cost-effectiveness analysis, but rather described them statistically. Jobs were categorised as formal and informal pre- and post-intervention. A formal job was defined as being involved in an occupation where the employee received a monthly salary. An informal job was working for a daily wage. Assessment of experience of violence was obtained from participants giving the answer ‘yes’ when asked whether they had experienced an incident of violence in interviews conducted pre- and post-intervention. The recall period for the experience of a violent incident was not included in the data for the pre-intervention stage. For the post-intervention stage, the recall period was same as the intervention period for the individual learner. Learners who did not respond to the surveys (pre and/or post) were categorized as ‘not reported’. The association between educational attainment and gender, age, job outcomes and experiences of violence before and after the ALS intervention was determined by performing a Pearson’s chi square test. To estimate the effect size, Cohen’s d effect size was calculated for the sample size for a statistical power of 80% and type-I error of 5% [17].

A cost analysis for the intervention was conducted from the service provider perspective. We included the costs borne by BRC during the recruitment process and the implementation and maintenance of the intervention. These costs were taken from the audited financial reports of the organization for the years 2015–18. The costs that were identified as being explicitly attributable to the ALS intervention were directly allocated and labelled as “Direct cost” (Table 1). This was followed by identification of the overhead costs from the budget. Those were the costs that were partially attributable to the ALS program, and were allocated based on their monthly time contribution to ALS related activities. These were labelled as “Shared costs” and included the salaries of supporting staff in proportion to the time spent in the ALS intervention compared to their total work time. This cost allocation was ascertained by listing the work responsibilities of the staff members and then shortlisting those activities that were related to ALS. Finally, an estimate of the monthly time spent on ALS-related activities was made by each individual staff member. The administrative cost and the planning and development cost which are also included in “Shared Costs” were identified on the basis of the involvement of staff members in these fields and its relation to the implementation, running and upkeep of ALS.

**Table 1:** Description and categorization of cost items

Cost categories	Description
Maintenance of Centre	Establishment and maintenance of youth learning centre -Monthly rental for the premises -Utilities costs (electricity, water, and upkeep) Accessories -Laptop and printer -Furniture and fixtures
ALS Intervention	ALS intervention and related activities -Community mapping for recruitment -Advertisements during recruitment process -Conducting literacy test before enrolment -Educational sessions -Study materials and stationery for students -Preparation of student’s portfolios for the examination -Transportation cost and refreshments on the day of examination -Salary of ALS instruction managers
Psychosocial Intervention	Screening and assessment of learners for their psychosocial needs -Profiling -Interviews -Focus group discussions -Home visits Psychosocial interventions -Case management through individual and group counselling -Psychoeducation sessions -Welfare assistance -Salary of Psychosocial workers
Salary of staff	Salaries of staff -Project supervisor -Program coordinator -Learning, Monitoring &Evaluation staff
Project Development	Development and evaluation of the project -Capacity building of staff -Monitoring and evaluation
Administrative Cost	Administrative cost -Administrative staff (Executive manager, Finance manager, Bookkeeper) -Project support costs (BRC office rent, supplies, utilities, and maintenance)

All costs are presented in 2018 price level [18]. Finally, the costs were converted to USD using Purchasing Power Parity (PPP) exchange rates [19]. The costs presented in the results were rounded to the nearest $10.

In the cost-effectiveness analysis (CEA) the ALS program was compared to a ‘do nothing approach’ where no educational intervention was implemented. As the design of the intervention is a one-group pre-post evaluation there was no equivalent group for comparison, so a counterfactual scenario was hypothesized based on contextual research. In a pre-intervention state in this setting, a school drop-out is likely to be unemployed, involved in paid or unpaid low-skilled work, or be involved in criminal activities [9]. We assume that a young person who was not in school and had not enrolled in an ALS program would not increase his or her educational attainment in terms of passing the A&E exam. Apart from the need for educational input, the ALS A&E exam is only available to students enrolled in an ALS Centre, and there are none in Bagong Silang apart from the one run by BRC. We do acknowledge that a similar outcome could be produced if a school drop-out returned to formal schooling, but this comes at a cost and is further discussed below. Therefore, in this study, a post-intervention state can only be achieved by taking an equivalence exam.

Costs and outcomes of the intervention are compared to the do-nothing scenario using an incremental cost-effectiveness ratio (ICER) that shows the extra cost for one extra positive outcome (pass in A&E exam). We also report the cost of the intervention per learner. To investigate the uncertainties in our results, two ambiguous parameters were identified and one- and multi-way sensitivity analyses were performed. Shared cost was estimated based on the self-reported time spent by the staff working for ALS and in the sensitivity analyses we varied this estimate by ± 50%. Further, the outcomes of attaining a pass in the ALS A&E examination at Elementary and Secondary level were considered equal in this study. In the sensitivity analyses, we only considered a pass at the secondary level as a successful outcome, which reduced the rate of successful outcome from 41 to 34%. IBM SPSS Statistics version 26 were used for all statistical analyses [20].

## Results

Of the total 239 learners enrolled in the intervention over the study period, 157 were children (12–17 years); 65 females and 92 males. Of the 82 adults (18 years old or more), 30 were females and 52 males. While everyone received the educational intervention only 22% of learners were found to need additional psychosocial interventions (Table 2).

**Table 2:** Background characteristics of the ALS learners (n = 239) and their outcomes

Characteristics	A&E exam			p-value
	Pass	Not pass	Total	
Age				0.18
Child (age < 18 years)	55 (60%)	102 (69%)	157 (66%)	
Adult (age > = 18 years)	36 (40%)	46 (31%)	82 (34%)	
Gender				0.8
Male	54 (60%)	90 (60%)	144 (60%)	
Female	36 (40%)	59 (40%)	95 (40%)	
Pre-intervention experience of violence				0.02
Yes	13 (14%)	40 (27%)	53 (22%)	
Not reported	78 (86%)	108 (73%)	186 (78%)	
Intervention				0.02
Education only	78 (86%)	108 (73%)	186 (78%)	
Education + Psychosocial	13 (14%)	40 (27%)	53 (22%)	
Post intervention education status				
Basic literacy	0 (0%)	5 (3%)	5 (2%)	
Elementary	17 (18%)	34 (23%)	51 (21%)	
Secondary^a^	80 (82%)	109 (74%)	189 (77%)	
Post intervention Job status^b^				0.006
Informal	20 (47%)	52 (72%)	72 (63%)	
Formal	23 (53%)	20 (28%)	43 (37%)	
Post intervention experience of violence				0.34
Yes	14 (15%)	30 (20%)	44 (18%)	
Not reported	77 (85%)	118 (80%)	195 (72%)	

ALS data on demographics and outcomes 2015–2018

^a^Include six learners who were initially enrolled at elementary level. They passed the elementary level and continued education to secondary level and later passed secondary level

^b^Missing values = 124

### Educational outcome

The A&E exam was held every year within six months of the end of the yearly intervention cycle. A total of 97 (41%) pass outcomes in the exam was divided between 91 learners (Cohen’s d effect size = 0.26). At the elementary level, where 51 learners enrolled, 17 secured a Pass grade in the A&E exam. 6 learners in this group continued their studies at secondary level and all of them secured a Pass at that level. Of the 189 learners who enrolled directly at secondary level, 74 secured a Pass in the examination.

Information on employment after participating in the intervention was available for 115 learners up to 31^st^ January 2020. A significant association (p-value = 0.006) in likelihood of having a formal job was noted for those who had passed the A&E exam.

### Experience of violence

The learners who reported on their experience of violence before enrolment in the intervention were also followed up until 31^st^ of January 2020. Pre-intervention experience of violence was found to be an unfavourable factor in attaining a pass in the A&E exam. This association was significant (p-value = 0.02). No significant association was found between the results in the examination and post-intervention violence experience (p-value = 0.34).

### Cost analysis

The total cost for the intervention during the period of January 2015 to September 2018 was 371,110 US dollars. The educational sessions of ALS accounted for 25% of the total cost, while the psychosocial intervention accounted for 14% of total cost. The total overhead costs (Shared cost) were almost the same as the Direct cost. The average cost of the intervention per enrolled learner (n = 239) was $1550 (Table 3).

**Table 3:** Overview of intervention cost and cost effectiveness

	Cost in USD
Direct cost	
Centre establishment and maintenance	21,140
Accessories	21,140
Educational sessions	21,790
Salary of Instruction manager	70,680
Psychosocial interventions	50,820
Salary of psychosocial worker	24,580
Shared cost	
Salary of supporting staff	60,540
Project development	30,290
Administrative cost	97,200
Total	371,110
Average cost per learner	1550
ICER	3830

*ICER* Incremental cost-effectiveness ratio

Considering a total pass score of 97, and total cost of intervention $371,110, this gives an incremental cost-effectiveness ratio (ICER) of $3830/pass in A&E Elementary or A&E Secondary. First one-way sensitivity analysis evaluated for uncertainties related to shared cost, average cost for learners enrolled varied from $1170 at the lowest level to $1940 at the highest level and the ICER varied from $2880 to $4770. In the second one-way sensitivity analysis where only a pass at Secondary level was considered to be a positive outcome the ICER increased to $4640. Finally, for the multi-way analysis the ICER for a pass at secondary level varied from $3490 to $5790 (Table 4).

**Table 4:** Sensitivity analysis

			Sensitivity analysis of average cost and ICER		
			Lower limit	Base case scenario	Upper limit
Model 1	Shared cost^a^	Average cost^d^	$1170	$1550	$1940
		ICER	$2880	$3830	$4770
Model 2	Single outcome^b^	ICER		$3830	$4640
Model 3	Multi-way analysis^c^	ICER	$3490	$3830	$5,90

^a^One-way sensitivity analysis to examine uncertainty in shared cost when varied ± 50%

^b^One-way sensitivity analysis to examine uncertainty if Pass in ALS A&E at Secondary level was the only positive outcome

^c^Multi-way analysis to examine uncertainty for both shared cost and single outcome combined

^d^Average cost per enrolled learner

## Discussion

This study investigated the outcomes, the cost, and the cost-effectiveness of the ALS intervention in Bagong Silang. The outcome of the intervention could be measured in terms of the success rate of learners passing the A&E examination at both elementary as well as secondary level. The national average of ALS learners passing the A&E exam was 30% and in this respect, this particular ALS intervention at Bagong Silang was found to be 11 percentage points more effective [8].

Besides the short-term outcome of the success rate in the A&E examination, another outcome measure of ALS was the job status of 115 learners who were followed up after their completion of ALS. Approximately 60% reported that they were employed in the informal sector and 40% in the formal sector. A significant unadjusted association was found between passing the exam and getting formal jobs, which suggest that a learner who passes the A&E exam is more likely to get a formal job. A 2018 report by the World Bank states that, in the Philippines, an individual who has enrolled at ALS and passed an A&E is twice as likely to get a formal job as someone who has not passed the exam [8]. Since job status is a long-term outcome, and information is not yet available for 50% of the learners, who finished the course only recently, we have not yet drawn conclusions about the effectiveness of ALS for job outcome in this report. The staff of BRC are at present trying to collect more follow-up data. We will reevaluate the intervention when this follow-up data is available.

The benefits of education for work prospects have been shown in other settings. For example, Kienzl et al. found that finishing high school in the USA is associated with a reduced risk of unemployment compared to non-completers [21]. However, this relation is time dependent; a person who completes high school with a shorter drop out period (less than four years) is more likely to have a positive economic outcome than a person who takes longer [21]. Since the age of most learners in the ALS program was 16–19 years of age, suggesting a short drop out period, we expect that the intervention will have a positive effect on future employment rates and further improve the cost-effectiveness of the ALS intervention and thus “the social return of investment”.

An aspect of the success of the ALS intervention that is harder to measure is whether it can reduce the exposure of the participants to experiences of violence. The available data concerning violence before and after the intervention did not show any striking change. However, the data on experience of violence are difficult to evaluate. Experience of violence is likely to have been under-reported among the enrollees [25% of those who had pre-intervention violence experience passed the A&E while 42% those who did not reported such experiences passed A&E (Table 2)]. UNICEF has reported that 80% of youths aged 13–24 years in the Philippines had experienced some form of violence in their lifetime, whether in the home, school, workplace, community or during dating [22]. To address the challenge for young people in Manila in terms of exposure to violent, BRC’s ALS intervention has a psychosocial component designed to enhance the physical and mental welling of young people at risk, which we see as a strength of this intervention. The impact of violence goes beyond physical consequences. Toxic stress from experiencing violence can negatively impact the wellbeing and intellectual development of young people [23].

It is known that high prevalence of violence has a negative impact on the people living in slums like Bagong Silang in terms of perceived and actual safety, school attendance, and businesses opportunities. Therefore, an ALS intervention with an effective psychosocial component is likely to result in more self-esteem, self-mastery, and self-control for the participants, and also diminished behavioral/emotional problems and violence [8]. In addition to addressing violence prevention, the psychosocial interventions in this program were designed with an intention to address risk factors that were the reasons behind low attendance of learners and prevent them from dropping out.

ALS interventions are an important way of helping young people to become better qualified for coping with adult life. However, such interventions must be financed, and in discussions about finance it is important to have an evaluation of what they might cost. Until now, cost analysis has not been routinely undertaken for evaluation of such interventions. The cost of the ALS intervention discussed here was found to be $1550 per enrolled learner, with an ICER of $3830. The overhead costs accounted for a huge portion of the total cost, which means that a high proportion of money is not spent directly in project but rather in administration. For many donors, the overhead ratio is an important indicator for the efficiency of a non-governmental organization. However, a high overhead does not mean that an intervention is not cost-effective. We believe that the high overhead cost reflects on the need of higher investment for initial organizational development in resource limited settings. The proportion for overhead can be expected to decrease in long run, especially if a higher number of learners can be enrolled.

The ICER for ALS was found to be $3830/passed A&E Exam. As there is no internationally accepted value of improved education, or of social interventions targeting out-of-school youths, it is difficult to establish whether the intervention studied was cost-effective. Comparing our results with those of similar interventions from similar contexts could indicate whether this was an efficient intervention. Unfortunately, all reports of comparable studies found in the literature were performed in high income countries, which poses a challenge for comparison. Kendall et al. conducted an economic evaluation of an alternative learning initiatives for out of school youths in United Kingdom and found that the average cost per learner enrolled was $7860 and the ICER for high school certificates obtained was $12,500 [24]. Another study undertaking a cost-effectiveness analysis done by Hollands et al. in the USA evaluated four different education programs targeting youths who had already dropped out of school. The average cost for learners enrolled in this study ranged from $15,750 to $20,890 and the ICER for extra high school completer ranged from $83,680 to $228,200 [25]. Based on the results of the comparative studies, the ALS intervention in Bagong Silang was found to be more cost-effective than the other informal education programs.

Since the published studies are of doubtful relevance to the situation in Manila, a more useful way of determining the worth of the investment is to compare it with the cost of school education in the Philippines. There is no tuition fee for primary and secondary education in government run public schools in Philippines. Budgets for maintenance and operating expenses are prepared at school level, which pose a challenge to calculate cost per student enrolled. Therefore, yearly tuition fee for private schools were referred. At elementary level (1–6 years) and secondary level (7–12 years) combined, the cost ranges from $54,160—$111,500 in 2019, which is around $4510–9220 per student per year [26]. The average cost of ALS per student per year in Bagong Silang was estimated to be $1550, which is 1/3 to 1/6 of the cost of attending a private school.

This study includes some parameters with uncertainties that may affect the estimated ICER. We performed two “one-way sensitivity analysis” and one “multi-way sensitivity analysis” and although this tended to increase the ICER, it remained in the lower range of prior studies and the cost of attending private schools in the Philippines. Another parameter of uncertainty in our model is the assumption of zero positive outcome in a counterfactual scenario of the ALS intervention, which is based on our best estimate. To check for the robustness of our assumption we investigated how many participants would have to pass the A&E exam in the counterfactual situation for the intervention under study to have the same ICER as the study by Kendall et al. The results show that 67/239 participants (i.e., 28%) would have to manage to pass the A&E exam without the ALS intervention, a number that we consider unrealistic as the national pass rate for ALS learners is 30%. This shows the range of uncertainty under which our conclusions about the cost-effectiveness hold and, we would argue, makes a strong point for the credibility of our conclusion. We therefore consider that the ALS intervention is cost-effective from a service provider’s perspective in achieving a higher level of education, which is a crucial step for youth empowerment, compared to a ‘do nothing approach’. However, this should not be an argument for students for dropping out of formal education and participating in non-formal education instead. As education in a formal setting offer more opportunity of engagement in the learning process, it plays an important role in developing non-cognitive skills in young people. Such skills are crucial for realising desirable life outcomes thus offering a sustainable solution to poverty alleviation [10, 27]. Dropping out from schools is very common for at-risk youths living in Bagong Silang. These young people often need to work to earn money, and even though public schools are free, the learners are unlikely to follow the regular curriculum successfully as they may miss many classes or fall asleep in class due to fatigue from jobs. In addition, the public schools in the slums in the Philippines are not always a safe haven for the students and are often burdened by youth gangs. It is hard to keep the good teachers in violence-prone neighborhoods. This touches on the issues of inequality, as poverty itself forces children and young adults to work and thereby forgo formal education, resulting in them being less able to compete in the future labor market. Therefore, we consider ALS a reasonable investment on behalf of at-risk young people living in slums in Manila if they are dismissed/drop out from the public schools, as other educational options are scarce.

We are aware that the self-recruited samples may differ from samples drawn from the general population. A previous study by Jensen et al. [5], shows that their target population (random sampling from Bagong Silang) has similar social demographics as our sample and therefore, has no influence on the effect size of the intervention as measured in the current study. We therefore do not consider significant differences in baseline. However, one potential factor that can affect our results is the motivation and attitude of the youth who are “self-recruited” for ALS, which might compromise the external validity. Since this ALS is not just an educational program, but a youth empowerment program with an integrated psychosocial framework which aimed to promote self-efficacy, we are unable to control for these effects (motivation etc.) on the results.

### Strengths and limitations

A strength of this study was that this youth empowerment program followed the guidelines and curriculum set by the government, which facilitated a comparison with the national data. Another strength of our study is a good sample size which allows estimation of small effects (Cohen’s d effect size = 0.26). A limitation is that we did not have a control group with which we could make a direct comparison. However, we considered that the results could be reliably compared with a ‘do nothing alternative’ for young people who did not attended BRC’s ALS intervention, as none of them would be able to get a pass in the examination, and there would be no cost associated with that outcome. We acknowledge that a similar outcome could be obtained if an out- of- school youth benefits from the intervention in another non-formal program or returns to a formal school. However, based on our contextual research we consider this unlikely. In any case, the results would still indicate the value of ALS interventions although not necessarily indicating the cost-effectiveness of the ALS intervention under study.

In this study, our emphasis for the analysis was on the immediate/short-term outcome of attaining higher level of education. Occupation is another determinant of socioeconomic status, and knowledge and skills are important factors determining job opportunities. Skills for a job can be gained through informal means as well. However, we assume that in the long run, completing secondary education would have better outcomes. Data on experience of violence is collected by a simple yes/no question, which is another limitation of this study. Future studies should use a standardized questionnaire to evaluate the impact of intervention on experiences of violence. Further, robust quasi-experimental study design such as interrupted time series which utilize segmented regression analysis are suggested for evaluating interventions where having a control group is not possible.

The ALS intervention is also expected to have wider societal benefits beyond the learner’s better employment/income status and violence prevention. Taking a societal perspective in the economic evaluation could potentially have unmasked some interesting results and could have guided decision makers within the government for future investments. An important step involved when considering a societal perspective is to include opportunity costs of the participants, i.e., the individual cost for attending ALS (e.g., reduced work income). It is suggested that taking an ingredient approach for cost analysis would have facilitated a study from a broader perspective [25].

### Conclusion

From a service provider’s perspective, the ALS for out of school youths with an ICER of $3830 per passed A&E Exam was found to be cost-effective compared to a ‘do nothing approach’. We hope that there will be long-term benefits that will improve the cost-effectiveness of the intervention, such as better job prospects, poverty reduction and reduction in experience of incidences of violence, as well as a change of safety perception in the community. Further research will be needed to evaluate these broader and long-term social and health benefits of ALS. We conclude that the intervention was a good investment on behalf of underprivileged youths living in the urban slum of Bagong Silang.

## Data Availability

The data that support the findings of this study are available from DIGNITY and from Balay Rehabilitation Centre, Manila, Philippines. Restrictions apply to the availability of these data, which were used under license for the current study, and are therefore not publicly available. Data are however available from the authors upon reasonable request and with permission of DIGNITY and Balay Rehabilitation Centre.

## Abbreviations

A&E: Accreditation and Evaluation exam
ALS: Alternate Learning System
BRC: Balay Rehabilitation Centre
CPI: Consumer Price Index
CEA: Cost Effectiveness Analysis
ICER: Incremental Cost-Effective Ratio
ISCED: International Standard Classification of Education
PI: Psychosocial Interventions
PPP: Purchasing Power Parity
UNESCO: United Nations Educational, Scientific and Cultural Organization
WHO: World Health Organization
